# Supplementary material for: Optimizing Detection of Kidney Transplant Injury by Assessment of Donor-Derived Cell-Free DNA via Massively Multiplex PCR
Source: J Clin Med. 2018 Dec 23;8(1):19. doi: 10.3390/jcm8010019 (PMC6352163; doi:10.3390/jcm8010019)
Supplement: Supplementary file 1 [file jcm-08-00019-s001.pdf]

Table S1. Performance characteristics for different dd-cfDNA cut-offs.

	**dd-cfDNA Cut-offs**					
	0.6%	0.8%	1.0%	1.2%	1.4%	1.6%
Sensitivity (95% CI)	95.2% (87.8–100)	93.5% (85.0–100)	88.7% (77.7–99.8)	85.5% (73.2–97.8)	80.7% (66.9–94.5)	69.4% (53.2–85.6)
Specificity (95% CI)	59.6% (51.7–67.6)	66.3% (58.7–74.0)	72.6% (65.4–79.8)	79.5% (73.0–76.0)	82.8% (76.7–88.9)	86.9% (81.4–92.3)
PPV (95% CI)	44.0% (38.8–49.2)	48.1% (42.0–54.2)	51.9% (44.7–59.2)	58.2% (49.7–66.7)	61.0 (51.6–70.3)	63.8% (52.8–74.8)
NPV (95% CI)	97.4% (93.4–100)	96.9% (92.8–100)	95.1% (90.5–99.7)	94.3% (89.7–98.9)	92.8 (88.0–97.6)	89.5% (84.5–94.5)

Table S2. Multiple groups comparisons.

dd-cfDNA

Comparison	Z	P.adj
**AR - BL**	**5.711116**	**0**
**AR - OI**	**4.569421**	**2.00E-05**
BL - OI	0.13599	0.891829
**AR - STA**	**6.746807**	**0**
BL - STA	1.107485	0.804253
OI - STA	0.644728	1

eGFR

Comparison	Z	P.adj
AR - BL	-1.17381	0.460946
AR - OI	-1.95735	0.15092
BL - OI	-1.15746	0.247085
**AR - STA**	**-8.65195**	**0**
**BL - STA**	**-9.08226**	**0**
**OI - STA**	**-5.19251**	**1.00E-06**

Significant (adj. $p < 0.0001$) results are bolded. The test to compares dd-cfDNA and eGFR for AR vs. Non-AR was a Kruskal-Wallis test.

Table S3. Cohort breakdown into for-cause and protocol biopsy for AR versus Non-AR.

**Status**	**Biopsy_Reason**	**Total**	**Median**	**Low**	**High**	**Mean**	**SD**
AR	For cause	25	2.04	0.09	23.9	3.85	4.81
	Protocol	13	3.56	0.12	23.4	6.16	6.44
Non-AR	For cause	78	0.645	0.02	6.54	1.08	1.27
	Protocol	101	0.27	0.03	6.78	0.80	1.28

Table S4. Descriptive statistics dd-cfDNA as a function of ABMR/TCMR

	**Count**	**Median**	**Low**	**High**	**Mean**	**SD**
ABMR	16	2.22	0.12	23.9	5.55	7.64
Both	12	2.56	0.09	8.8	4.00	3.21
TCMR	10	2.69	1.01	9.77	3.96	3.11

Table S5. Histology report for all active rejection cases.

**Histology Report**	**dd-cfDNA Assay Result**	
	**<1% dd-cfDNA**	**>1% dd-cfDNA**
ABMR	0	1
ABMR + acute bTCMR	0	1
ABMR + acute TCMR IA	0	4
ABMR + acute TCMR IIB	0	2
ABMR + bTCMR	3	2
ABMR + chronic active bTCMR	0	5
ABMR + chronic active TCMR IA	0	1
ABMR + chronic active TCMR IB	1	0
ABMR + chronic active TCMR II	0	2
ABMR + chronic active TCMR IIA	0	1
acute ABMR	0	1
acute TCMR IA	0	2
bABMR + acute TCMR IA	0	2
bC4d-positive ABMR + TCMR-IA	1	0
chronic active ABMR + bTCMR	0	2
chronic active TCMR IA	0	1
chronic active TCMR IB	0	3
TCMR IA	0	2
TG + cABMR	0	1

Table S6. Descriptive statistics for dd-cfDNA as a function of donor type.

**Group**	**Donor_type**	**Total**	**Median**	**Low**	**High**	**Mean**	**SD**
	Living Related	1	0.97	0.97	0.97	0.97	N/A
AR	Living Non-related	2	4.88	1.39	8.37	4.88	4.94
	Deceased Non-related	35	2.41	0.09	23.9	4.74	5.59
	Living Related	14	0.265	0.02	3.89	0.67	1.09
Non-AR	Living Non-related	75	0.65	0.05	6.78	1.17	1.54
	Deceased Non-related	90	0.405	0.06	6.3	0.75	1.02

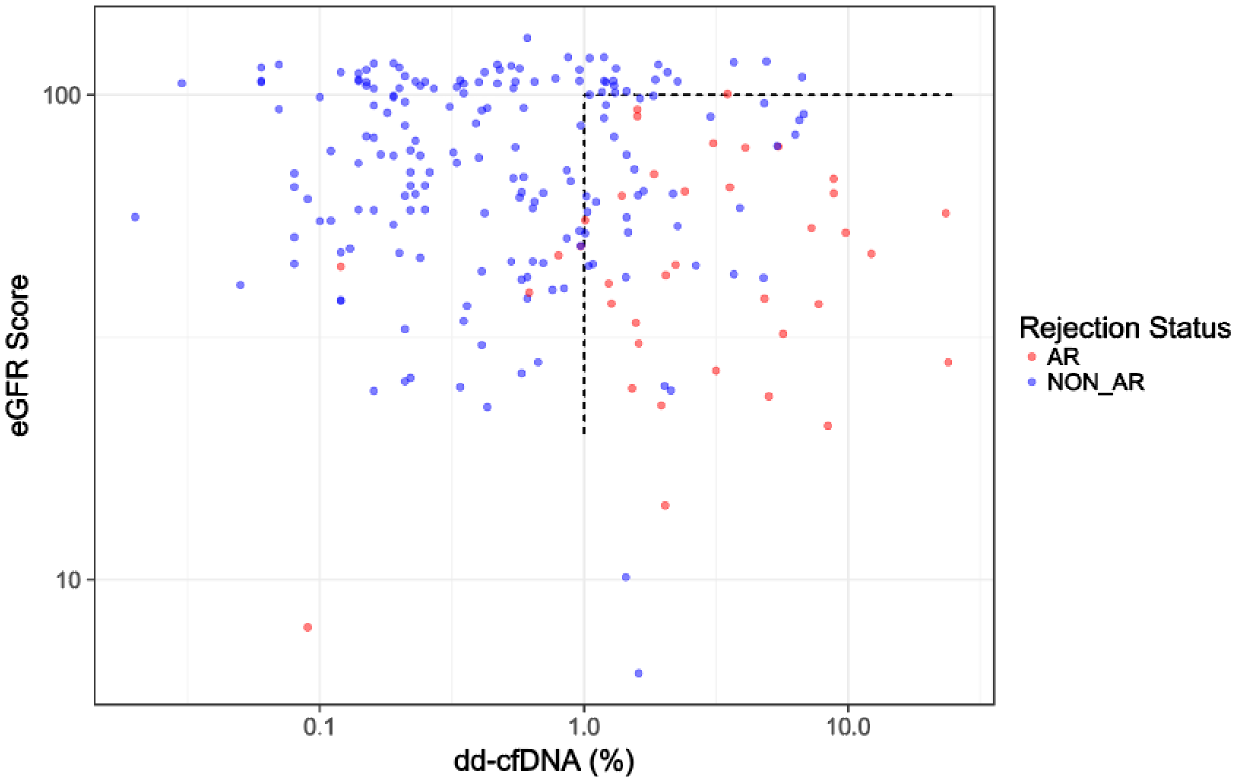

Figure S1. dd-cfDNA and eGFR in biopsy matched samples.

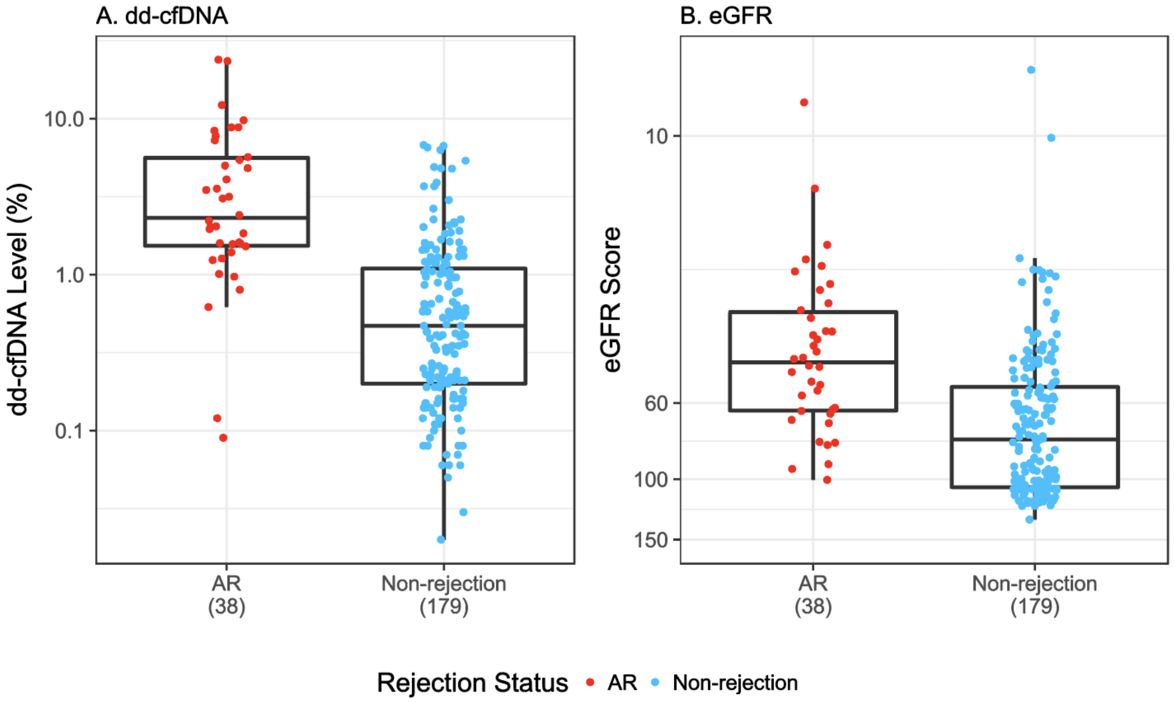

Figure S2. Discrimination of active rejection by dd-cfDNA versus eGFR in AR versus non-AR.

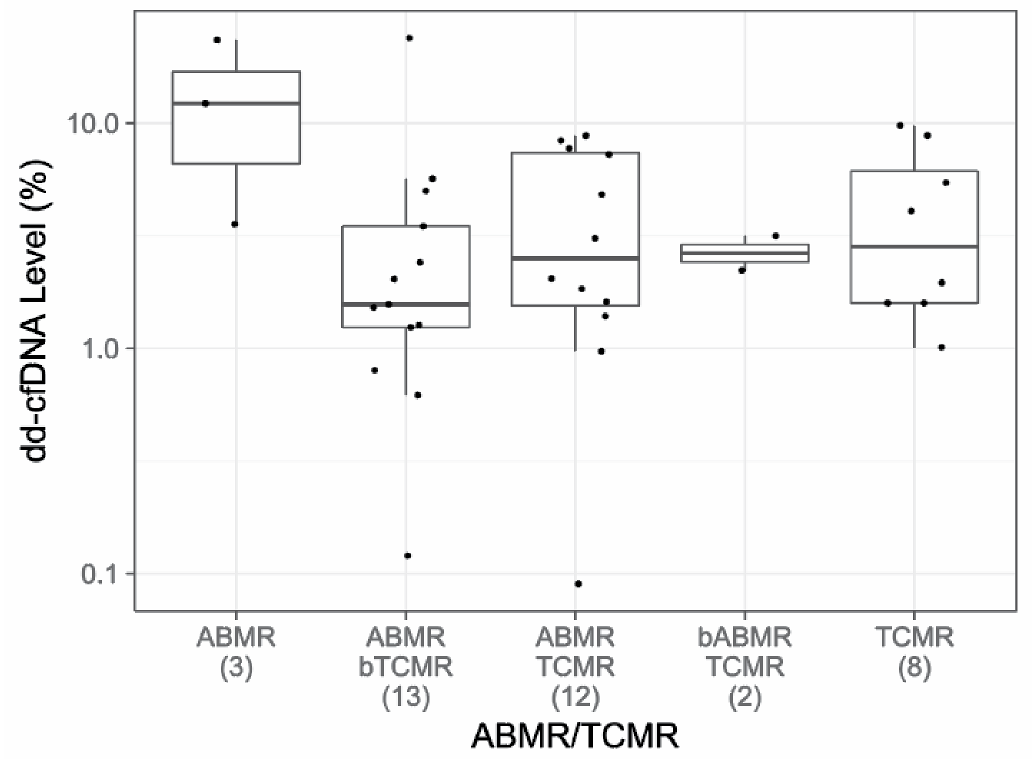

Figure S3. Percent dd-cfDNA by ABMR/TCMR status.
